# Wolff on duties of esteem in the law of peoples

**DOI:** 10.1111/ejop.12593

**Published:** 2020-09-27

**Authors:** Andreas Blank

**Affiliations:** ^1^ Department of Philosophy Universitätsstr Alpen‐Adria Universität Klagenfurt Klagenfurt Austria

## Abstract

The role that the desire for self‐worth plays in international relations has become a prominent topic in contemporary political theory. Contemporary accounts are based on the notion of national self‐worth as a function of status; therefore, the desire for national self‐worth is seen as a source of anxiety and conflict over status. By contrast, according to Christian Wolff, there exists a duty to take care that both one's own and other political communities deserve to be esteemed. In his view, this duty is grounded in the duty of self‐perfection because the qualities for which communities deserve to be esteemed are those that promote the self‐perfection of individuals. From this perspective, he argues that duties of esteem toward political communities should not be seen as an outcome of power relations but rather as an outcome of the fulfillment of functions of the law of peoples. Wolff's line of argument draws attention to the possibility that anger over a violation of the desire for honor could be mistaken for an expression of specific culture‐specific sensitivities, while what really may be expressed is a diverging interpretation of the implications of dependence in international relations.

## 1 INTRODUCTION

In the past two decades, a rich literature in political theory has arisen over the question of how the desire to uphold self‐worth influences international relations. What is perhaps most surprising about this literature is that concepts such as honor, which had seemed to be obsolete, have turned out to be illuminating tools for analyzing how certain cultural attitudes influence attitudes toward one's own people and toward other peoples (Donelan, 2007; O'Neill, 1999). Disregarding feelings of honor is a source of international conflict because honor cultures—Arabic countries and the countries in the territory of the former Soviet Union are frequently mentioned examples—by their nature are prone to conflict. As Jörg Friedrichs has recently put it: “Honor cultures are troubled because honor is ambivalent. It is neither intrinsic nor extrinsic, but internalized. At the same time, it is neither entirely alienable nor entirely inalienable, but contestable” (Friedrichs, 2016, p. 69). It is well documented how much the competition for honor leads to anxieties concerning the status of one's own nation, and how much status anxiety influences decisions in power politics (Paul, Welch Larson, & Wohlforth, 2014), especially decisions concerning warfare (Lebow, 2010, 2015; Renshon, 2016; Wohlforth, 2009). Contemporary political literature has discussed various culture‐specific forms that the desire for national self‐worth can take. It is widely held that the contemporary Western world has moved from honor cultures to dignity cultures, where self‐worth is defined through the possession of basic rights and liberties. A further way of constituting self‐worth—the desire to save face—is understood to be an attitude characteristic of many Asian countries (Friedrichs, 2016, pp. 68–79). According to these lines of thought, taking seriously the problem of insensitivity toward culture‐specific feelings of self‐worth may significantly improve international relations. And because such intercultural approaches to international relations take into account differences between cultural traditions, they are regarded as alternatives to neo‐Hegelian theories of unconditional recognition (as articulated, for example, in Honneth, 1995, 2012; Wendt, 2003).

From a historical point of view, the resurgence of these issues in contemporary political thought is striking because early modern cultures were honor cultures and questions of honor, reputation, respect, esteem, and glory were prominent topics in early modern political theory. However, read from the perspective of their early modern counterparts, the contemporary approaches to the role of honor in international politics seem to overlook two issues that were central to early modern discussions and still are central to present‐day political practice. The first issue concerns the connection between honor and status; the second issue concerns the connection between independence and sovereignty. As to the first issue, contemporary political theory seems to take it to be a conceptual truth that honor reflects nothing but a position in a social hierarchy. By contrast, early modern natural law theorists understood honor as an expression of esteem for naturally good qualities, both of individuals and of nations. It seems to be a worthwhile enterprise to ask whether such conceptions of esteem between nations could offer an alternative to conflict‐laden conceptions of national self‐worth. As to the second issue, status‐oriented approaches to honor seem to imply that dependent political communities cannot make the same claims to self‐worth as the communities on which they depend. In political practice, this intuition is expressed by withholding full diplomatic rights to the representatives of dependent communities. By contrast, early modern natural law theorists considered the possibility that even dependent communities can fulfill the natural goals of civil government and therefore have all the diplomatic rights that derive from sovereignty. There may be situations where such intercultural accounts of international relations ascribe difficulties in international communication to cultural differences whereas what is really at stake are the diverging interpretations of the implications of relations of political dependence.

In what follows, I will substantiate this suggestion through an analysis of the treatment of the question of esteem for political communities in the thought of Christian Wolff (1679–1754). Focusing on Wolff may draw attention to the possibility of thinking about honor and esteem in international relations in an alternative, non‐competitive way. Honor, it may turn out, could not only be understood, as it is sometimes done (for instance, in Wolf, 2011), as a function of the position in a social hierarchy; it could also be understood as a function of esteem for the fulfillment of the natural goals of political communities. And in contrast to a position in a social hierarchy, the degree to which a community fulfills natural goals of government cannot be contested merely on grounds of power relations. The value of fulfilling natural goals of government does not consist in surpassing other nations, but rather, assisting other nations in their ability to fulfill these goals can be supportive of the ability of one's own nation to fulfill these goals. Therefore, Wolff takes duties of esteem toward one's own nation to be compatible with duties of esteem toward other nations.

Focusing on Wolff will also bring to light an implicit presupposition of the specifically modern way of thinking of esteem between political communities—a certain, debatable conception of sovereignty—as well as some possibilities to think about duties of esteem for political communities in an alternative way once these debatable presuppositions have been replaced by an alternative conception of sovereignty. Wolff diverges in this respect from a line of thought that goes back to Jean Bodin (1530–1596). According to Bodin, sovereignty requires independence from external interference and therefore cannot be divided between different political actors (Bodin, 1576, I. 8). While Wolff shares the independence criterion, he regards sovereignty as bundles of rights that can be distributed between different political actors. Even if a political community can lose some sovereignty rights, it still retains sovereignty in those rights that fulfill the natural goal of political communities and that remain independent of external interference. If so, then the duties of esteem deriving from the sovereign character of a political community remain intact.

## 2 SELF‐PERFECTION AND DUTIES OF ESTEEM TOWARD ONE'S OWN PEOPLE

Famously, Wolff is part of the early modern person‐of‐the‐state tradition that treats peoples, like other associations, as legal persons (Wolff, 1749, § 2). According to this tradition, peoples are the bearers of rights and the subject of the ascription of actions.^1^ In Wolff, the commitment to this tradition is expressed, for example, in his treatment of the liability of a people for the actions of its political leadership: As Wolff argues, just as an action of someone can be imputed upon another individual who would have been obliged to express dissent with the action, and who was in the position to express this dissent, but failed to do so, so can an action of the magistrate be imputed upon the entire people whose members had been obliged to object to the action of the magistrate, if they had been in a position to do so and failed to do it (Wolff, 1749, § 314). At the same time, Wolff also is part of the German law tradition that regards peoples as special cases of associations in the sense of a multiplicity of individuals standing in certain personal and legal relations to each other. Wolff takes a people (gens) to be “a multitude (multitudo) of individuals associated in a political community (civitas) for the purpose of achieving a certain goal with conjoined forces” (Wolff, 1749, § 35; see Wolff, 1740–1748, p. 8; §§ 4–5). As Otto von Gierke has pointed out in his work on the German law of associations, regarding associations as multitudes of individuals makes it plausible to regard associations as bearers not only of rights and liabilities but also of esteem and honor (von Gierke, 1868, pp. 365; 391; 517).

Keeping the presence of both aspects of Wolff's notion of a people in mind lends plausibility to his strategy of construing duties between political communities as analogous to duties between individuals. Generally, he claims that political communities owe to each other exactly what individuals owe to each other (Wolff, 1749, § 156)—a claim that would be unintelligible if political communities were understood only as legal persons. Using an equivocation on the term “person” may be problematic because Wolff alludes to the theory of legal fictions when he regards peoples as persons. No matter how useful such fictions may be for bringing out intuitions concerning what would be rational to do in certain situations, legal fictions also introduce a strong dissimilarity between peoples and individuals. This is so because, by definition, legal fictions assume something that we know is contrary to what is true.^2^ For instance, Andrea Alciato maintains that a legal fiction is “a disposition of the law that goes against the truth in a possible situation and is introduced for a just cause” (Alciato, 1551, p. 8). Invoking legal fictions is particularly problematic when it comes to transferring duties from the level of individuals to the level of peoples. We know that legal fictions do not have feelings, hence also no feelings of honor and sensibilities toward esteem. This is why it is problematic to assume that everything that we know about the esteem‐related natural needs of individuals could be transferred to peoples, understood as legal persons.

To aggravate this problem, Wolff develops his account of the duties of esteem toward other political communities in the context of a cosmopolitan theory of relations between peoples. As in his conception of the nature of peoples, the theory of legal fictions plays a significant role in Wolff's conception of the civitas maxima—the political community that encompasses all political communities. Francis Cheneval has made a strong case for the usefulness of the theory of legal fictions for Wolff's cosmopolitanism (Cheneval, 1999, 2002, pp. 132–202; see also Greenwood Onuf, 1994). Wolff makes use of the Roman‐law conception that the presumed consent of an individual who has not consented gives rise to a quasi‐contract that brings the same legal obligations with it as a regular contract (Wolff, 1740–1748, p. 5; § 504). In Wolff's view, it is the consideration of what peoples would consent to if their actions were fully rational that justifies establishing rules of positive (“voluntary”) international law that can be enforced on the basis of democratic decisions taken by the majority of peoples (Wolff, 1749, § 19).

But again, it is not clear whether a theory of legal fictions helps much in establishing duties of esteem toward other peoples. Wolff understands duties of esteem toward other peoples as duties of humanity, not as duties of justice (Wolff, 1749, § 251, note). Duties of humanity, for Wolff, belong to the realm of imperfect duties, that is, to the realm of duties that give rise to moral evaluation but not to enforcement (Wolff, 1740–1748, p. 1; § 859). Therefore, these duties do not belong to the realm of what Wolff calls the “voluntary law of peoples” that is constitutive of the civitas maxima (Wolff, 1749, sig. b3v‐c1r). If legal fictions do any useful work in Wolff's conception of cosmopolitan duties, then they ground strict, enforceable duties. Hence, the concept of a universal society understood as a legal fiction does not contribute much to understanding the foundations of imperfect duties such as duties of esteem toward other peoples.

Luckily, these problems can be remedied by considering Wolff's conception of peoples as concrete multitudes—that is, as multitudes of individuals with natural needs and esteem‐related feelings. From this perspective, what connects duties of esteem toward individuals with duties of esteem among communities is not just an equivocation on the term “person” but rather causal connections between beliefs about political communities, beliefs about single individuals who belong to these communities, and the role that these beliefs play in the fulfillment of self‐related duties. As it will turn out, these connections are mediated through the causal role that the degree of perfection of a political community—its ability to fulfill the goals of civil government—has on the degree of self‐perfection of individuals, both of those who belong to this community and of those who belong to other communities. Due to the influence of the perfection of political communities on individual self‐perfection, our duties to perfect ourselves involve duties to perfect political communities, both our own and other communities. And in so far as duties of esteem are implied by duties of perfection, duties of esteem—toward other individuals, toward one's own political community, and toward other political communities—these duties are causally linked to self‐related duties. In this way, duties of esteem toward political communities can be regarded as extensions of duties of esteem toward others that, ultimately, are grounded in duties of self‐perfection.

Some conceptual clarifications will be helpful. As Wolff defines it, “[p]erfection is coherence in variety … Coherence is the tendency toward realizing one and the same goal” (Wolff, 1736, § 503). This structure can be found in organic bodies, such as when the parts of an eye tend toward the production of a clear and distinct retinal image. It also can be found in artifacts, such as when the parts of a clock tend toward showing the exact time. It also can be applied to the life of individuals: “life, in so far as it denotes a complex of free actions, is called perfect if all of these actions tend toward a goal toward which also the natural actions tend” (Wolff, 1736, § 503). Wolff describes the perfection of the soul as the coherence between the uses of the different powers of the soul (Wolff, 1740–1748, p. 1; § 201) and points out that, due to the divergent final causes that trigger them, vicious actions never lead to full coherence in acting—neither to coherence between free actions nor to coherence between free and natural actions (Wolff, 1740–1748, p. 1; § 504, note).

In Wolff's view, the notion of perfection can also be applied to peoples. As he defines it, the perfection of a people consists in its aptitude for realizing the goal of civil government (Wolff, 1749, § 29), and the goal of civil government consists in common well‐being (Wolff, 1740–1748, p. 7; § 11). As he explains, “[t]he common well‐being of a political community consists in that it abounds in what contributes to the necessities, the comfort and the pleasures of life, that it provides conditions sufficient for the happiness of individuals, that everyone is secure from the injury of others and the violence of external enemies, and that the community is strong enough to offer resistance in these respects” (Wolff, 1740–1748, p. 8; § 9). Wolff is clear that the entire eighth part of the Jus Naturae should be regarded as an explication of this abstract characterization of the goal of civil government (Wolff, 1740–1748, p. 8; sig. B4r‐v). For the notion of the perfection of a people, it is therefore highly relevant that he understands a people not only as a legal person but also as what he calls a “composite entity” (ens compositum) whose parts are individuals (Wolff, 1749, § 29, note). As he analyzes it, in order to realize the goals of civic government, citizens in their many ways of living must be able to fulfill their functions correctly (Wolff, 1749, § 29, note). And in order to be able to do so, it is the task of political constitution‐making to provide a legal framework that defines these functions in a way that reflects external conditions (Wolff, 1749, § 30, note). As in the case of an individual, the degree of perfection of a people thus consists in the degree to which a multiplicity of actions contributes to realize goals that are naturally good.

Wolff's considerations concerning the notion of perfection are closely connected with his notion of natural obligation, both on the level of individuals and on the level of peoples. As Wolff holds, humans are obliged by their nature to carry out intrinsically good actions (Wolff, 1744, p. 1; § 127). As he argues, there is a close analogy with obligations that arise from human conventions. On the side of obligations that arise from conventions, “[t]he person obliges us to carry out actions, who connects with them a motivation for wanting to do them” (Wolff, 1744, p. 1; § 127). But this is something that not only human beings can do through their actions; human nature can do it, as well: “the essence and nature of humans and things has conjoined a motivation with intrinsically good and bad actions” (Wolff, 1744, p. 1; § 127). For this reason, an obligation exists to carry out actions that tend toward one's perfection (Wolff, 1744, p. 1; § 128). The obligation in question is described as a “natural obligation,” that is, an obligation that has sufficient reason in human nature (Wolff, 1744, p. 1; § 129). Everything that contributes to fulfilling the duty of self‐perfection falls under this heading, including duties toward others that are instrumental for self‐perfection. As a matter of common experience, Wolff points out, everyone needs the help of others for self‐perfection (Wolff, 1744, p. 1; § 220)—certainly a point that applies to all activities that can be improved through feedback from others. Therefore, he holds that there is a natural obligation to promote perfection with conjoined forces (Wolff, 1744, p. 1; § 221). Since others can contribute more to our perfection the better they are themselves, the obligation to promote perfection with conjoined forces implies an obligation to contribute as much as we can and as much as is needed for the perfection of others (Wolff, 1744, p. 1; §§ 222; 232). Again, Wolff takes this to be a precept of natural law, because it follows from human nature (Wolff, 1744, p. 1; § 223).

Duties toward political communities—both to one's own community and to other political communities—can be regarded as special cases of these other‐related duties. Since the goals of civil government defined by natural law are naturally good, a people has a natural obligation to develop the ability to pursue these goals; and since the perfection of a people consists in its perfection to pursue the goals of civil government, a people is naturally obliged to perfect itself (Wolff, 1749, § 35). This duty has several aspects: One aspect consists in the duty to cultivate self‐knowledge, since a people needs to know what can prevent its perfection (Wolff, 1749, § 40); as Wolff explains, this can be regarded as a duty of a people toward itself (Wolff, 1749, § 40, note). The same duty applies to those who govern the people (Wolff, 1749, § 41), and Wolff is clear that the relevant knowledge must include information concerning the different regions and natural talents, habits and life‐forms of their inhabitants (Wolff, 1749, § 42).

The duty to perfect one's own condition also includes esteem‐related duties, both with respect to other individuals and with respect to political communities.^3^ As Wolff defines it, esteem (existimatio) is a judgment of others about our perfection, especially about the acquired perfections of our souls (Wolff, 1740–1748, p. 1; § 538), that is, our moral and intellectual virtues (Wolff, 1740–1748, p. 1; § 548). By using this definition of esteem, Wolff adopts one of two distinct concepts of being held in “good esteem” (existimatio bona) that were debated in the early German Enlightenment. One of these concepts can be described as a juridical concept, the other as an ethical concept (Achenwall & Pütter, 1750, §§ 96–99). According to the juridical concept, a citizen who has not been convicted of any wrongdoing is in the possession of full civic dignity, understood as the possession of full civic rights. The right to being held in good esteem in the juridical sense consists in the right of being presumed not to have violated the laws in the absence of contrary evidence.^4^ By contrast, the ethical concept of good esteem denotes judgments concerning moral and intellectual virtues. Taking acquired perfections of the soul to be the proper objects of esteem, as Wolff does, places his conception of esteem unambiguously on the side of the ethical concept of esteem.

In line with the ethical concept of esteem, Wolff understands honor as the set of acts through which we signify our judgment about the perfection of others (Wolff, 1740–1748, p. 1; § 550). In this way, he distinguishes between esteem and acts that express esteem; still, honor is closely bound to esteem—that is, to acts of the intellect, not to blind responses to status. Accordingly, Wolff embraces a normative conception both of esteem and honor. In his view, only the virtues, not positions in civil life, make someone worthy of esteem and honor (Wolff, 1740–1748, p. 1; § 552). Likewise, we have a duty to contribute to our own reputation (fama) (Wolff, 1740–1748, p. 1; § 554), where reputation is understood as the common discourse concerning someone's intellectual and moral dispositions (Wolff, 1740–1748, p. 1; § 553).

Wolff's analysis of duties of esteem and honor with respect to others is closely analogous to his analysis of self‐related duties of esteem and honor. In his view, we have the duty to esteem (Wolff, 1740–1748, p. 1; § 647) and honor (Wolff, 1740–1748, p. 1; § 648) others as much as they deserve. Again, he is clear that this duty relates to natural honor, not to civil honor, which he takes to consist often only in opinion (Wolff, 1740–1748, p. 1; § 648, note). This normative conception implies that others deserve esteem and honor only proportional to the degree of their perfection. Plausibly, the duties of esteem and honor with respect to others are a special case of our duties of knowing others, which, as we have seen, is regarded as a necessary means for self‐perfection. Wolff regards esteeming others as much as they deserve as something that is useful both for others and for ourselves: “The praise through which we express esteem ignites the ardor in progressing further in what is good, reconciles the minds of others with us, and excites the emulation of others” (Wolff, 1750–1753, p. 5; § 479, note). Esteeming the good personal qualities of others is thus meant to increase the perfection of those who are esteemed, to increase the perfection of those who take those who are esteemed as role models, and to improve our personal relations with others.

Also, the esteem‐related duties toward one's own people derive from the self‐interest of individuals—the interest that individuals take in the reputation (fama) of their people. As Wolff notes, what holds true for the majority of a people usually is predicated upon its single members; in this sense “it cannot be avoided that what is predicated upon all belongs, as it were, to each individual” (Wolff, 1749, § 43); and these dynamics are all the more worthy of attention as they apply not only to true beliefs but also to prejudices (Wolff, 1749, § 43, note). This is why individuals have the duty to contribute as much as they can to the reputation of their people (Wolff, 1749, § 44); and this can be done by cultivating moral and intellectual virtues, as well as by abstaining from the pathologies of esteem that, in Wolff's view, are characteristic for the republic of letters in Germany. As he argues, the widespread mutual detraction between German scholars and their adulation for academic life outside of Germany (not an unknown phenomenon nowadays) are detrimental for the reputation of the German people; and it is a self‐refuting practice because these scholars would participate in the reputation of their compatriots (Wolff, 1749, § 44, note).

Forms of competition between scholars that take the form of mutual detraction thus are detrimental for the reputation of a whole community and thereby also for the reputation of those who compete for esteem. On the contrary, letting other scholars of the same community enjoy the reputation that they deserve increases the reputation in which this community is held, and thereby the reputation of each of its members. This gives an interesting hint at how non‐competitive practices of esteem could enhance the esteem in which all members of a community are held. This line of argument shows why we may be wrong to assume that we enhance the esteem in which we are held by trying to diminish the esteem in which other members of our political community are held. On the contrary, for Wolff there is not only a duty to care about one's own reputation (fama) but also a duty to care about the reputation of one's own people (Wolff, 1749, § 46). As Wolff argues, scholars play a prominent role as guardians (conservatores) of the reputation of their people, which is why it is a serious offense if scholars detract anything from the reputation of their people (Wolff, 1749, § 46, note). In his view, these dynamics illustrate the general insight that the usefulness of any association depends on the reputation in which it is held (Wolff, 1749, § 46, note).

An analogous argument can be built for duties to uphold the glory of one's own people, where Wolff understands glory (gloria) as the “consistent praise of the good and experienced” (Wolff, 1749, § 47). Consequently, the glory of a people as “praise of the good and experienced about the perfection of intellectual and moral virtues of those who belong to the people” (Wolff, 1749, § 48). In Wolff's analysis of how glory can be attributed to a people, both his theory of legal persons and his account of peoples as concrete multitudes come together:

Glory is attributed to a people primarily and per se in so far as it is regarded as a single person, to whom one's own actions that depend upon intellectual and moral virtues are attributed; but it is also attributed in so far as the praise that belongs to individuals due to their actions or deeds that are regarded as belonging to individuals. (Wolff, 1749, § 48, note).

Again, natural obligations with respect to the glory of a people arise from the natural goodness of the qualities that constitute perfection. As Wolff argues, the glory of a people depends on perfection; a people is obliged to perfect itself; hence, a people is obliged to strive to deserve glory (Wolff, 1749, § 48). Also, individuals have not only to strive to deserve glory themselves, but also that they relate their actions to the glory of their people (Wolff, 1749, § 48, note).

At the same time, Wolff reminds us of the pathological forms that the desire for glory can take, both on the level of individual and on the level of peoples: Striving for glory as the ultimate end constitutes the vice of ambition (ambitio) (Wolff, 1740–1748, p. 1; § 570; Wolff, 1749, § 48, note); striving for unmerited honors is the vice of arrogance (arrogantia) (Wolff, 1740–1748, p. 1; § 566; Wolff, 1749, § 48, note); the desire to be credited with higher perfection than is actually there is the vice of vanity (vanitas) (Wolff, 1740–1748, p. 1; § 583; Wolff, 1749, § 48, note); and the overly strong desire for honor shows a lack of the virtue of modesty (modestia) (Wolff, 1740–1748, p. 1; § 563; Wolff, 1749, § 48, note). Emphasizing that the glory of peoples always must be bound to moral virtues indicates that the goal of glory is not to surpass other peoples but rather to fulfill the demand of natural goodness as well as possible. This leaves open the possibility that other peoples enjoy the same degree of glory—a possibility that is, as we shall presently see, crucial for Wolff's conception of duties of esteem for other peoples.

## 3 SELF‐PERFECTION AND DUTIES OF ESTEEM TOWARD OTHER PEOPLES

So far, the outlines of Wolff's non‐competitive conception of duties of esteem within one's own political community should have become clear. In his view, fulfilling duties of esteem based upon intellectual and moral virtues are instrumental in upholding the esteem in which one's own people is held, which in turn reflects positively upon the esteem in which the individuals belonging to this community are held; and making a people deserving of esteem increases the positive influence that the individuals belonging to this community can play in their own self‐perfection. Perhaps now it is not so surprising to see that, contrary to the view that striving for honor in international relations is essentially a matter of competition, Wolff holds that practices of esteem toward other political communities should be non‐competitive, as well. As he maintains, “[w]e must esteem and praise each people as much as it deserves” (Wolff, 1749, § 186), where esteem for peoples should be proportional to their perfection (Wolff, 1749, § 186, note). He argues that because these duties hold in the relation among individuals (Wolff, 1740–1748, p. 1; § 647), they also hold with respect to duties toward peoples (Wolff, 1749, § 186). Is this an instance of an inconclusive equivocation on the term “person”?

Not only, since Wolff also exploits an analogy between the influence of knowing the qualities of other individuals on self‐perfection and the influence of knowing the qualities of other peoples on self‐perfection. On the level of individuals, he regards it to be a duty to know the perfections and imperfections of others, since this leads to insight into the perfections and imperfections that human beings are capable of and insight into the means that they are using for self‐perfection (Wolff, 1740–1748, p. 1; § 193). He also adds that there is a duty to consult what others have found out about perfection and imperfection of soul and body and the use of human powers in self‐perfection, because this also belongs to the means of self‐perfection (Wolff, 1740–1748, p. 1; § 196). The connection between self‐perfection and knowledge of others indicates a sense in which “the entire human species is united by very close bonds, in such a way that one human being can be of use to others and that one needs the assistance of others” (Wolff, 1740–1748, p. 1; § 195). Wolff holds that there is a duty that falls upon travelers and the erudite whose task it is to make known what is praiseworthy in people because thereby they provide exemplars that are worthy of imitation (Wolff, 1749, § 186, note). Thus, duties of esteem toward other peoples are grounded in duties of self‐perfection. As he comments: “The duties toward others cohere in such a way with duties toward oneself that they have a mutual influence on each other” (Wolff, 1749, § 186, note).

At the same time, Wolff's conception of duties of esteem toward other peoples involves a comparative aspect that makes room for disesteeming other peoples—namely exactly when they do not achieve the goals of civil government. This becomes clear in his considerations concerning the notion of a civilized people. In his view, being civilized consists in cultivating intellectual virtues in such a way that also moral virtues are cultivated. This is why he reckons with the possibility of what he calls “semi‐barbarous” peoples that cultivate intellectual virtues without thereby cultivating moral virtues; the members of these peoples are disfigured by an inflated esteem for intellectual achievements and a lack of self‐knowledge concerning their moral faults (Wolff, 1749, § 53, note). In this sense, Wolff's conception of justified esteem for peoples differs from the conception of unconditional recognition. Wolff's conception of duties of esteem for other peoples cannot be understood as advocating recognition in the sense of acceptance of the life forms of other peoples, no matter how dysfunctional their political, legal and administrative institutions may be.

But Wolff would also not acquiesce with merely expressing disesteem for the aspects of the life forms of less civilized peoples that fail to fulfill the natural goals of civil government. Rather, he holds that there is also a duty to enhance the esteem in which other peoples are held. As it turns out, this is a duty that derives from the insight that not only the perfection of one's own people but also the perfection of other peoples can be instrumental in fulfilling duties of self‐perfection. Wolff maintains that each people must contribute to the perfection of other peoples in every way that it can, if other peoples cannot procure it for themselves (Wolff, 1749, § 166). As he makes clear, these duties toward other peoples imply duties toward one's own people: As Wolff argues, because a people cannot contribute much to rending other peoples more perfect without perfecting itself, it has to perfect itself with a view to fulfilling duties to other peoples (Wolff, 1749, § 180).

Wolff is aware of the practical difficulties involved in any attempt to contribute something to the perfection of other people; he notes that an objection could be raised that he is extending duties toward other peoples too far, and that at best it is possible to contribute something to the perfection of neighboring peoples (Wolff, 1749, § 185, note). In response to such an objection, Wolff contends that practical obstacles only suspend the duty to contribute all that is needed to the perfection of other nations, but such obstacles do not abolish it (Wolff, 1749, § 185, note); and he argues that geographical distance cannot make duties toward other peoples less cogent since these duties arise from human nature (Wolff, 1749, § 185, note). But he concedes that taking care of neighboring peoples sometimes may be the most effective method to achieve something for the most remote peoples (Wolff, 1749, § 185, note). And what can be done even across great distances is to give good examples; for this reason, he holds that there is a duty to give good examples to other peoples (Wolff, 1749, § 181). From this duty follows that a people must make itself be worthy of being imitated by other peoples, and that peoples should imitate those peoples that are worthy of being imitated (Wolff, 1749, § 184); and what Wolff has in mind is rational, well‐informed imitation, in contrast to nebulous prejudices about peoples (Wolff, 1749, § 184).

The resulting view could be called a cooperative conception of duties of perfection—the point is not to surpass other peoples with respect to perfection but rather to increase the perfection of all peoples as far as it can be done. This conception of duties of perfection also implies specifically esteem‐related duties. As Wolff puts it: “Each people must take care that also other people deserve glory” (Wolff, 1749, § 185). He offers two arguments. The first argument regards this insight as an application of the principle that each people owes to other peoples what it owes to itself without neglecting duties to itself (Wolff, 1749, § 156). However, this argument derives from the problematic assumption that all natural obligations that hold between individuals can be transferred to peoples, understood as persons (Wolff, 1749, § 156). The second argument avoids this shortcoming. This argument makes use of the insight that each people must contribute everything that it can and that is needed to the perfection of other peoples (Wolff, 1749, § 166); this implies the duty to take care that other peoples deserve glory since glory depends on perfection (Wolff, 1749, § 49). In this way, Wolff's conception of the duty to make other peoples more deserving of esteem is closely analogous to his conception of the duty to make one's own people more deserving of esteem. Both kinds of duties ultimately derive from the observation that we can enhance our own perfection only with conjoined forces, and that the more possibilities of conjoining forces for this purpose, the better the prospects of enhancing our own intellectual and moral qualities.

## 4 POWER, SOVEREIGNTY AND ESTEEM IN DIPLOMATIC RELATIONS

Do Wolff's arguments for duties of esteem toward other peoples imply that considerations of power become entirely irrelevant for assessing such duties? The answer has to be a cautious “no.” Wolff holds that there are some esteem‐related duties toward other peoples that can—and even should—express power relations; at the same time, he insists that there are esteem‐related duties that cannot be a function of power relations. This becomes clear in his discussion of duties of esteem in diplomatic relations.

As to the former group of duties, Wolff accepts the view that rules of precedence laid down in protocol can solve a practical problem that arises from power differences. In his view, due to the equality of natural rights, natural qualities do not naturally justify any prerogative between peoples, as little as they do between individuals, where differences in moral and intellectual virtue do not give rise to an inequality of rights (Wolff, 1749, § 237). In this sense, Wolff holds that there is a natural equality of rights between peoples (Wolff, 1749, § 238). However, he argues that peoples have the right to give up rights through contracts (Wolff, 1749, § 239). Contracts concerning protocol in meetings of delegates from different peoples are a good example for giving up rights in this way (Wolff, 1749, § 241). And in matters of protocol, Wolff notes, it may be simply a matter of prudence to concede precedence to the more powerful (Wolff, 1749, § 241).

However, Wolff is clear that matters of precedence and other matters of prerogative do not affect those esteem‐related duties that are based on the exertion of sovereignty rights. This becomes clear in his discussion of how different forms of alliances affect sovereignty rights. As he explains, equal confederations are confederations in which the parties promise to each other the same or equivalent things, for instance in trade alliances where the goods exchanged are of equal value (Wolff, 1749, § 395). From a normative point of view, he holds that, if equal services are in the power of the parties, then these parties should form equal confederation, unless there are exceptional reasons for deviating from this rule, such as temporary necessities (Wolff, 1749, § 397); this is so because there is no natural duty to offer services for free in situations where an equivalent compensation can be given (Wolff, 1740–1748, p. 4; § 268). Evidently, exchanging services of equal value does not amount to a diminishing of sovereignty rights. As Wolff argues, this also holds for those unequal confederations where the rights that are conferred upon the stronger party do not belong to sovereignty rights (Wolff, 1749, § 399). But he is also clear that some unequal confederations diminish sovereignty rights, for example when they involve contractual restrictions concerning the rights of warfare. He holds that if one people can offer more services than another people, then unequal confederations are in conformity with the law of peoples (Wolff, 1749, § 404). In his view, such situations could even be used to fulfill the natural duty to contribute everything that is possible and needed for the perfection of other peoples; and unequal confederation become illegitimate only when the allies do not fulfill the duties of natural law (Wolff, 1749, § 404, note).

Wolff uses here a notion of sovereignty that, in one respect, shares something with the tradition initiated by Bodin but in another respect fundamentally departs from this tradition. Wolff agrees with Bodin that sovereignty requires independence from external interference. However, while for Bodin the independence criterion implies that sovereignty by its essence is indivisible, Wolff holds that sovereignty consists in “the right to do those things that are required to promote the public good in a political community” (Wolff, 1740–1748, p. 8; §§ 35; 60). Since many things are required for the public good, and there is a right to do each of them, sovereignty can be understood as a complex of many rights (Wolff, 1740–1748, p. 8; § 61). Wolff makes use of this conception when he holds that each part of sovereignty can be transferred either with respect to exercise or with respect to substance (Wolff, 1740–1748, p. 8; § 39). This distinction is particularly relevant for his analysis of what happens in unequal alliances that bring with them a diminution of sovereignty rights. Even where legitimate unequal alliances diminish sovereignty rights, he maintains, they do not abolish it. If only the exercise of sovereignty rights has been transferred, then the substance of sovereignty remains undivided (Wolff, 1749, § 400). But even splitting up the substance of sovereignty rights can take away the character of sovereignty from those rights that remain immune from interference from the outside (Wolff, 1749, § 401, note). For instance, “[a] tributary people does not lose sovereignty” (Wolff, 1749, § 411) since the duty to pay a tribute derives from a confederation contract that does not transfer other rights (Wolff, 1740–1748, p. 8; § 44). Moreover, Wolff argues that matters of prerogative are accidental and subject to change and therefore cannot affect the essence of sovereignty. And even if some sovereignty rights can be transferred with respect to substance, those rights that relate to the fulfillment of the goals of civil government that remain independent from external interference retain their sovereign nature (Wolff, 1749, § 250, note).

In either way, dependent political communities can retain sovereignty rights; and in Wolff's view, sovereignty rights give rise to duties of esteem that do not reflect power differences. Generally, Wolff holds that sovereignty implies equality of dignity and that the equality of dignity requires an equal display of signs of honor, independently of the prerogative of one people over another (Wolff, 1749, § 250, note). A special case of duties of esteem arising from equal dignity are duties of esteem in sending and receiving ambassadors. As Wolff argues, one of the sovereignty rights that always remains intact in unequal alliances is the right to send ambassadors (Wolff, 1749, § 1046). He regards this right as a natural right because it is grounded in several other natural rights of peoples: the perfect right of seeking offices of humanity (Wolff, 1749, § 170); the imperfect right of seeking to establish commercial relations (Wolff, 1749, § 189); and the imperfect right of seeking to establish perfect rights in the relations with other peoples through forming alliances (Wolff, 1749, §§ 370; 377). And although Wolff uses the generic term “legati,” which could refer to envoys of all kinds, his emphasis on the representative character (character repraesentativus) of legati indicates that what he has in mind are envoys that act in the name of a community that possesses sovereignty (summa potestas) (Wolff, 1749, § 1,055).^5^

Understanding the concept of representative character in this way also explains why Wolff regards equality of dignity to be central to the esteem‐related duties connected with the “legati.” As he argues, equality of the dignity involves the duty to choose persons of high social standing and impeccable reputation for the function of ambassadors, since doing otherwise would express contempt for the heads of the sovereign community to whom they are sent (Wolff, 1749, § 1050). Moreover, equality of dignity implies that the heads of the sovereign community who receive ambassadors have the duty to treat them honorably (Wolff, 1749, § 1051). Since this is a duty of natural law and, hence, remains valid even in situations of warfare, this duty applies even with respect to ambassadors sent by enemies (Wolff, 1749, § 1052). Likewise, Wolff takes it to be illegitimate to use defamation against ambassadors sent by an enemy since this involves a violation of duties of honor and thereby violates the rights of the sovereign community that has sent them (Wolff, 1749, § 1053). Also the duty to care for the security of ambassadors derives not only from the consideration that security is a prerequisite for the ability to fulfill the natural functions of ambassadors; it also derives from the consideration that violating the security of ambassadors would be contrary to recognizing the equality of dignity of sovereign communities (Wolff, 1749, § 1064). Esteem‐related duties in sending and receiving ambassadors thus fulfill a significant political function because they express the recognition of equal dignity grounded in the fulfillment of functions of civil government. Fulfilling duties of esteem in sending and receiving ambassadors thus is a special case of the duty to esteem other peoples as much as they deserve.

## 5 CONCLUSION

Now it should be clear that Wolff's conception of duties of esteem toward political communities is not only based on a problematic equivocation on the term “person” but also on considerations concerning the causal connections between self‐perfection, the perfection of one's own community and the perfection of other communities. On the level of individuals, Wolff offers a conception of esteem and self‐esteem that, in principle, allows for the possibility that everyone can be held in high esteem and develop high self‐esteem. This is so because the striving for perfection can be realized only by joining forces to contribute as much to the perfection of others as is needed and as is possible. These mutual duties of perfection involve mutual duties of esteem—duties of esteeming others as much as they deserve and duties of contributing as much as possible to other's deserving esteem (in contrast to deriving satisfaction from diminishing the reputation of others). This conception of duties of esteem can easily be extended to duties toward one's own political community and toward other communities, and this is exactly what happens in Wolff's discussion of these duties in the law of peoples.

Wolff thereby formulates a natural law‐based conception of duties of esteem in the law of peoples that give hints at how the problem of international competition for status could be solved. His conception of honor does not make esteem among peoples a function of power relations. It is comparative in the sense that it allows for assessing degrees in which political communities fulfill duties of the law of peoples. At the same time, the value of how well political communities fulfill these functions does not derive from being better than the average performance in a competition. Rather, the value of fulfilling such functions derives from the role that doing so has in meeting basic human needs; and (apart from matters of precedence) power is only relevant for the capability of meeting these needs. This way of thinking thus reduces the status anxiety experienced by dependent communities and lowers the potential for conflicts over status.

From this perspective, Wolff develops a compelling conception of the duties of esteem due to dependent political communities—those communities who have lost some of their sovereignty rights to more powerful communities. Wolff defends a conception of sovereignty that allows for geographically overlapping sovereignty rights. What from Bodin's perspective must look like a dissolution of sovereignty may bring serious advantages in political practice because it offers the theoretical foundations that would allow recognition of the functions of the law of peoples that dependent political communities still may be able to fulfill. Those functions of civil government that political communities still can exert independently from more powerful communities still count as an expression of sovereignty rights, with all the duties of esteem that these rights bring with them.

This seems to be a view that still is thought‐provoking when read from the perspective of present‐day intercultural approaches to international relations. To be sure, it may be a good idea not to give the representatives of a former superpower who cultivate an honor culture the impression that one takes them to be political actors of merely regional importance. And it may be a good idea not to let political actors from a face‐oriented culture lose their face. But there is also the possibility that resentful reactions may be taken to be an expression of culture‐specific sensibilities when in fact something different is at stake. If signs of esteem that are due to independent peoples are denied to political communities that do not possess independence but nevertheless are able to fulfill functions under the law of peoples, then this constitutes a violation of a natural right to esteem. If so, then there is a genuine possibility that the anger caused by a lack of esteem could be mistaken for an expression of culture‐specific sensitivities to matters of honor, whereas what really occurs is anger over the lack of recognition for the fulfillment of functions of the law of peoples. In such cases, greater caution not to offend honor‐ or face‐related sensitivities may not only be inefficient to avoid tensions; it may also exacerbate the feeling of being offended in one's natural rights.

## Endnotes

^1^
For a detailed history of this tradition, see Holland, 2017.

^2^
On legal fictions in early modern legal thought, see Maclean, 1992, pp. 101–103; König, 1998, pp. 144–155.

^3^
On Wolff's analysis of duties of esteem between individuals, see Blank, 2019.

^4^
On the role of this idea in the early German Enlightenment, see Hruschka, 2000.

^5^
On the notion of representative character in early modern natural theory theories, see Hofmann, 1974, pp. 182–186.
